# Temporal Trends in Mental Disorder Rates among Patients with Colorectal Cancer: A Comprehensive Analysis

**DOI:** 10.3390/jcm13133649

**Published:** 2024-06-22

**Authors:** Christoph Roderburg, Sven H. Loosen, Catherine Leyh, Andreas Krieg, Sarah Krieg, Markus Jördens, Tom Luedde, Karel Kostev

**Affiliations:** 1 Department of Gastroenterology, Hepatology and Infectious Diseases, University Hospital Düsseldorf, Medical Faculty of Heinrich Heine, University Düsseldorf, Moorenstrasse 5, 40225 Düsseldorf, Germany; sven.loosen@med.uni-duesseldorf.de (S.H.L.); catherine.leyh@med.uni-duesseldorf.de (C.L.); markus.joerdens@med.uni-duesseldorf.de (M.J.); tom.luedde@med.uni-duesseldorf.de (T.L.); 2 Department of General and Visceral Surgery, Thoracic Surgery and Proctology, University Hospital Herford, Medical Campus OWL, Ruhr University Bochum, 32049 Herford, Germany; andreas.krieg@klinikum-herford.de; 3 Department of Inclusive Medicine, University Hospital Ostwestfalen-Lippe, Bielefeld University, 33617 Bielefeld, Germany; sarah.krieg@mara.de; 4 Epidemiology, IQVIA, 60549 Frankfurt, Germany; karel.kostev@iqvia.com

**Keywords:** colorectal cancer, CRC, depression, anxiety, stress

## Abstract

**Background:** Colorectal cancer (CRC) stands as one of the most prevalent and burdensome malignancies worldwide. Similar to other cancers, CRC has been associated with the development of psychiatric diseases, including anxiety and depression. However, temporal trends in psychiatric disorders rates within CRC patients have not been investigated so far. **Methods:** The present study included 15,619 individuals with colorectal cancer and 78,095 propensity score-matched individuals without cancer, who were identified within the Disease Analyzer (IQVIA) database in Germany between 2005 and 2022. Cox regression analysis was conducted to assess the association between CHC and subsequent psychiatric diseases, including depression, anxiety disorders, and adjustment disorder, by period (2005–2010, 2011–2016, 2017–2022). **Results:** The 12-month cumulative incidence of any psychiatric disorder diagnosis in the CRC cohort increased from 6.3% in 2005–2010 to 8.2% in 2017–2022. The strongest increase was observed for reaction to severe stress and adjustment disorder (1.0% in 2005–2010 to 2.6% in 2017–2022). Notably, the strong increase in psychiatric disorders was not specific for cancer patients since a slight increase in psychiatric disorders was also observed in the non-cancer cohort. Regression analyses revealed that CRC was strongly and significantly associated with an increased risk of depression, anxiety disorders, reaction to severe stress and adjustment disorders, as well as any psychiatric disorder. Of note, the extent of the association was stronger in 2017–2022 compared to 2005–2010, clearly proving a “real” increase in the rates of psychiatric disorders over time. **Conclusions:** This study presents novel data from a large cohort of outpatients in Germany, providing strong evidence for an increase in psychiatric disorders in the recent years. These findings contribute to the existing body of literature and should trigger the recognition of psychiatric problems in cancer survivors.

## 1. Introduction

Colorectal cancer (CRC) stands as one of the most prevalent and burdensome malignancies worldwide [1,2]. According to the 2022 Global Cancer Statistics [3], CRC is the second leading cause of cancer-related mortality, with an estimated 903,859 cancer deaths worldwide in 2022. As the population rapidly ages, cancer incidence continues to rise. Over 65% of new cases occur in developed countries, with almost half of all new cases estimated to occur in Europe and the Americas. While much attention has rightfully been directed towards advancing treatment modalities and improving survival outcomes, the psychological toll of CRC, particularly its association with depression, has gained increasing recognition in recent years. Notably, such research is particularly important in view of the dramatic increase in the survival rates and survival times of CRC patients.

Physical and mental health problems, usually secondary to the cancer and its treatment, are experienced by many cancer survivors, including those with CRC [4,5,6,7]. Of note, the psychological impact of colorectal cancer is profound and multifaceted. Patients often experience a range of emotional responses, including depression, anxiety, fatigue, pain, and cognitive deficits [8,9,10,11,12]. Cancer survivors can experience these symptoms for more than 10 years after treatment [13]. A recent review article reported depression and anxiety in 13–25% of CRC patients [14], while other studies found high point prevalences of sleep disorders [15] and depression in 13–57% of CRC patients [16]. By conducting a review of 15 studies published between June 1967 and June 2018, Peng et al. reported a prevalence of depression among patients diagnosed with CRC ranging from 1.6 to 57% and anxiety ranging from 1.0 to 47.2% [17]. Moreover, the social and occupational disruptions caused by the disease [18] can lead to feelings of isolation and decreased quality of life. Understanding these psychological impacts is crucial for providing comprehensive care that addresses both the physical and mental health needs of CRC patients.

While ample research has underscored the prevalence of mental diseases among cancer patients, a comprehensive understanding of the temporal trends in depression rates within this specific cohort remains limited. By analyzing a large database of >10 million outpatients treated in 1293 general practices in Germany [19], our study seeks to bridge this gap by systematically examining the trajectory of depression incidence among colorectal cancer patients compared to non-cancer individuals, shedding light on potential contributing factors and implications for clinical practice and public health policies.

## 2. Methods

### 2.1. Database

This retrospective cohort study was based on data from the Disease Analyzer database (IQVIA). This data source has already been used in several previous studies focusing on depression and colorectal cancer [19,20,21,22], and it contains anonymous data on diagnoses, prescriptions, and basic demographic data from computer systems used in office-based practices [23]. The database contains approximately 3000 office-based practices in Germany. The sampling method for the Disease Analyzer database uses statistics from the German Medical Association to determine the panel design according to the specialist group, German federal state, community size category, and age of physician. It has previously been shown that the panel of practices included in the Disease Analyzer database is representative of general and specialized practices in Germany [23]. 

### 2.2. Study Population

**Figure 1:**
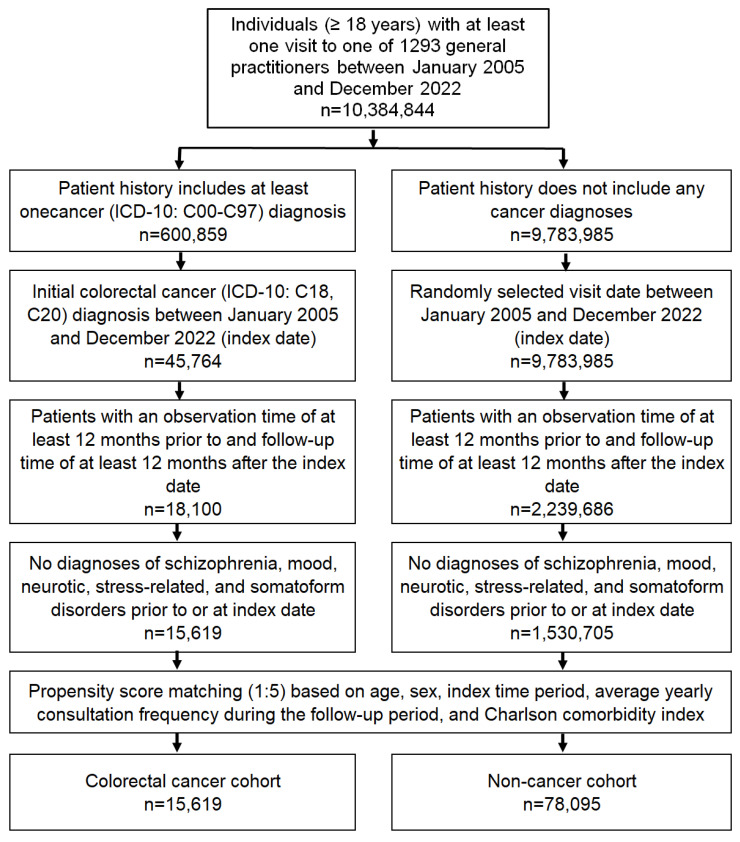
Selection of study patients.

This study included individuals aged ≥ 18 years with a CRC diagnosis (ICD-10: C18, C20) in 1293 general practices in Germany between January 2005 and December 2022 (index date; Figure 1). Further inclusion criteria were an observation time of at least 12 months prior to the index date and a follow-up time of at least 12 months after the index date. Patients with schizophrenia (ICD-10: F20–F25), mood (ICD-10: F30–F39), neurotic, stress-related, and somatoform disorders (ICD-10: F40–F48) documented within 12 months prior to or on index date were excluded. After applying similar inclusion criteria, individuals without cancer were matched to CRC patients using nearest-neighbor propensity score matching (1:5) based on age (±2 years), sex, index time (2005–2010, 2011–2016, 2017–2022), average yearly consultation frequency during the follow-up, and Charlson Comorbidity Index (CCI, without cancer). CCI is a weighted index that accounts for the number and severity of comorbidities and includes a wide range of comorbidities [macrovascular diseases, pulmonary diseases, gastrointestinal, liver, and renal diseases, diabetes, tumors, and AIDS] [24]. For the non-cancer cohort, the index date was that of a randomly selected visit between January 2005 and December 2022 (Figure 1). Standardized mean difference (SMD) was considered to examine the balance of covariate distribution between study cohorts after matching. In this study, we allowed a SMD of less than 0.1, indicating that adequate covariate balance was achieved [25,26].

### 2.3. Study Outcomes and Statistical Analyses

The outcomes of this study were the initial diagnoses of depression (ICD-10: F32, F33), reaction to severe stress and adjustment disorder (ICD-10: F43), and anxiety disorders (ICD-10: F41) during the 12 months following the index date as a function of CRC. The main study aim was, however, to evaluate the difference of psychiatric disease incidence after CRC diagnosis between three time periods (2005–2010, 2011–2016, 2017–2022). The 12-month cumulative incidence of psychiatric diseases in the cohort with and without CRC was studied with Kaplan–Meier curves in each of the three time periods. Finally, an univariable Cox regression analysis was conducted to assess the association between CHC and subsequent psychiatric diseases by period. Results of the Cox regression model are displayed as hazard ratios (HRs) and 95% confidence intervals (CIs). Due to multiple models, a *p*-value of <0.01 was considered to be statistically significant. Analyses were carried out using SAS version 9.4 (SAS Institute, Cary, NC, USA).

## 3. Results

### 3.1. Basic Characteristics of the Study Sample

In total, 15,619 individuals with colorectal cancer and 78,095 individuals without cancer were included in this study. Basic characteristics of the patients are summarized in Table 1. Mean age was 68.8 (standard deviation (SD): 12.4) years. Moreover, 44.4% of patients were female and 55.6% were male. Patients visited their GPs on average 8.2 times during the 12-month follow-up period. Due to a matched-pairs design, no significant differences were observable between both cohorts in terms of age, sex, GP visit frequency, Charlson Comorbidity Index and index period. Overall, 3782 of patients with colorectal cancer and 16,320 patients without cancer were available in the period of 2005–2010, 5882 with colorectal cancer and 29,367 without in the period of 2011–2016, and 6455 patients with colorectal cancer and 32,408 without colorectal cancer in the period of 2017–2022, respectively (Table 1).

### 3.2. Cumulative Incidence of Documented Psychiatric Disorders

**Figure 2:**
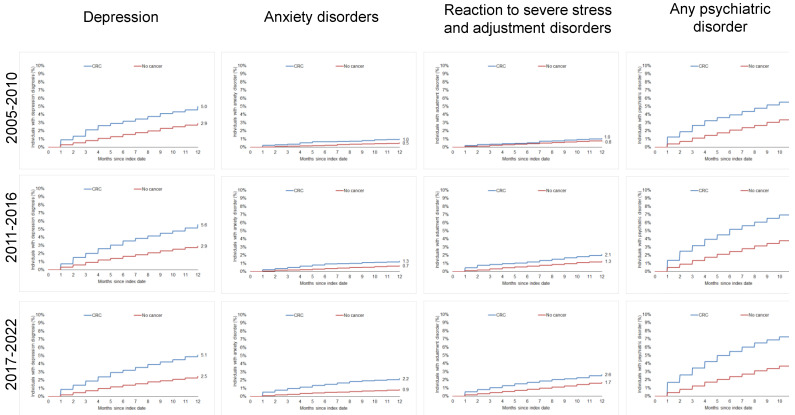
Cumulative incidence of psychiatric disorders in individuals with and without colorectal cancer by period.

The 12-month cumulative incidence of any psychiatric disorder diagnosis in the CRC cohort increased from 6.3% in 2005–2010 to 8.2% in 2017–2022. The strongest increase was observed for reaction to severe stress and adjustment disorder (1.0% in 2005–2010 to 2.6% in 2017–2022) followed by anxiety disorders (1.0% in 2005–2010 to 2.2% in 2017–2022). In contrast, this increase was less clear for the term “depression” (5.0% in 2005–2010 to 5.1% in 2017–2022; Figure 2). Notably, the strong increase in psychiatric disorders was not specific for cancer patients since an increase in psychiatric disorders was also observed in the non-cancer cohort (Figure 2).

### 3.3. Time Changes in the Association of CRC with Subsequent Psychiatric Disorders

In the regression analysis, CRC was strongly and significantly associated with an increased risk of depression, anxiety disorders, reaction to severe stress and adjustment disorders, as well as any other psychiatric disorder. However, the extent of the association was stronger in 2017–2022 compared to 2005–2010 ((any psychiatric disorder: HR = 1.64; 95% CI: 1.41–1.92) in 2005–2010 and HR = 1.90; 95% 1.72–2.10 in 2017–2022, depression: HR = 1.73 (95% CI: 1.45–2.07) in 2005–2010 and HR = 2.08; 95% 1.83–2.36 in 2017–2022, anxiety disorders: HR = 1.86 (95% CI: 1.24–2.79) in 2005–2010 and HR = 2.59; 95% 2.11–3.17 in 2017–2022, reaction to severe stress and adjustment disorders: HR = 1.31 (95% CI: 0.91–1.89) in 2005–2010 and HR = 1.55; 95% 1.31–1.84 in 2017–2022)) (Table 2), clearly proving a “real” increase in the rates of psychiatric disorders over time. 

## 4. Discussion

We show that the incidence rates of mental disorders among CRC patients have significantly risen since 2010. The strongest increase was observed for reaction to severe stress and adjustment disorder, while the increase was weaker for depression. Regression analyses confirmed that CRC was strongly and significantly associated with an increased risk of depression, anxiety disorders, reaction to severe stress and adjustment disorders, as well as any other psychiatric disorder. Of note, the extent of the association was stronger in 2017–2022 compared to 2005–2010, clearly proving a “real” increase in the rates of psychiatric disorders over time. 

To our knowledge, this study represents the first analysis on temporal trends in psychiatric disorders rates within CRC patients. In the past, ample research has underscored the prevalence of mental diseases among cancer patients. As an example, Massie et al. synthesized 88 studies, observing depression correlations primarily with oropharyngeal, pancreatic, breast, and lung cancers, ranging from 1.5% to 57% [14]. Conversely, colon and gynecological cancers, as well as lymphoma, exhibited lower prevalence rates (13–25%) [14]. Despite extensive efforts to evaluate depression among cancer patients, estimates vary widely. Walker et al. identified 66 relevant studies, but only 15 met quality criteria, reporting depression prevalence ranging from 5% to 16% in outpatients, 4% to 14% in inpatients, 4% to 11% in mixed settings, and 7% to 49% in palliative care [16]. Specifically, in patients with colorectal cancer, Peng et al. reported a prevalence of depression among patients diagnosed with CRC ranging from 1.6 to 57% and anxiety ranging from 1.0 to 47.2% [17]. The importance of such data for the care of cancer survivors cannot be overestimated. Depression and anxiety significantly impact health functions and mortality risk in cancer patients, with meta-analyses indicating up to a 25% higher mortality rate in those with depressive symptoms [16]. Chida et al.’s analysis of 165 studies revealed a higher mortality risk associated with depression in both community-based cancer survivors (relative risk (RR) = 1.34) and cancer patients (RR = 1.08). Another meta-analysis reported that depression predicts mortality, though not disease progression, in cancer patients, with estimates indicating a 26% greater mortality rate among patients with depressive symptoms and a 39% higher rate among those diagnosed with major depression [17]. Finally, a large prospective population-based study on elderly CRC patients explored the link between depressive symptoms and mortality up to 10 years post-diagnosis, revealing increased mortality rates among 1- to 10-year and 1- to 2-year CRC survivors with depressive symptoms [10]. Surprisingly, the suicide risk among elderly CRC patients is low (<0.2%), with no discernible differences based on primary tumor location [27]. Thus, our data once again underline the need for targeted screening and subsequent specific psychological care for patients with tumor diseases, such as colorectal carcinoma. Care by specially trained psycho-oncologists is required in this context according to international guidelines (e.g., American Cancer Society CRC Survivorship Care Guidelines). One model that could be used here would be to regularly examine all tumor patients in both outpatient and inpatient settings for the need for further diagnostics using a so-called distress thermometer test [28]. This test should then trigger further psycho-oncological interventions, if needed.

In the present study, we showed that the incidence rates of mental disorders among CRC patients have significantly increased since 2010. Several factors might contribute to this observed increase in mental disorder rates among CRC patients: First, an enhanced awareness and screening efforts for mental health issues, coupled with advancements in diagnostic techniques, might have led to higher detection rates of depression and anxiety among CRC patients over the recent decades. In this context, it is important to note that there is a growing acknowledgment within the medical community of the psychological impact of cancer diagnosis and treatment, prompting more thorough assessments of CRC patients’ mental health statuses. On the other hand, a decreasing stigma surrounding mental health issues may have encouraged CRC patients to seek help for psychological symptoms, leading to higher reported rates of depression and anxiety in the GP setting. Recently, the shift in CRC treatment approaches such as the introduction of more aggressive therapies or the refinement of surgical techniques has contributed to longer survival rates, meaning that more patients experience CRC as a chronic illness that might exacerbate psychological distress over time, leading to higher rates of depression and anxiety. Finally, it is important to note that more aggressive treatment strategies might provoke increased psychological distress among patients due to concerns about treatment efficacy, side effects, and prognosis. Further longitudinal research is needed to better understand the underlying factors driving the trend observed in our study and to develop targeted interventions to support the psychological well-being of individuals living with CRC. In this context, Luo et al. aimed at exploring existing survivorship interventions after CRC treatment, according to the American Cancer Society CRC Survivorship Care Guidelines, to provide evidence for such interventions. By analyzing thirty studies that met the inclusion criteria, the authors showed “mounting evidence for various interventions to improve distress/depression/anxiety”. Moreover, emerging evidence was found for the implementation of survivorship care plans to improve patient perceptions of care coordination [29].

Our study has several limitations that warrant consideration. Firstly, our reliance on ICD-coded diagnoses introduces uncertainty regarding the accuracy of these codes, potentially affecting the representativeness of absolute incidence values. Nonetheless, given the large patient cohort included, it is reasonable to assume that any coding errors would likely be distributed evenly across both study groups. Notably, the frequency of psychiatric disorders in our database seems rather low. In line with this, previous research has highlighted the discrepancy between administrative diagnosis documentation and actual patient antidepressant prescriptions, further complicating our analysis [27]. Moreover, the inherent limitations of database analyses, such as data incompleteness, prevent us from providing comprehensive information on individual disease stages, guideline-based therapies, or disease progression. Consequently, we cannot differentiate between metastatic and non-metastatic CRC cases. Additionally, the lack of data on tumor localization within the database is noteworthy, as the distinction between left-sided and right-sided CRC is crucial due to the differences in tumor characteristics, treatment responses, and prognoses. Furthermore, crucial socioeconomic and lifestyle-related factors, such as education, income, smoking, alcohol consumption, and physical activity, are absent from the Disease Analyzer database, precluding their consideration in our analysis. Finally, a retrospective study design does not allow conclusions to be drawn on causal relationships, only on statistical associations. Despite these limitations, the utilization of data from a large database instills confidence in the reliability and clinical significance of our findings.

In conclusion, this paper endeavors to provide a comprehensive overview of the current trends in mental disorders among colorectal cancer patients, offering insights into its dynamic nature and implications for clinical management and public health strategies. We aspire to pave the way for tailored interventions aimed at enhancing the holistic care and support of individuals with colorectal cancer.

## Figures and Tables

**Table 1:** Baseline characteristics of the study sample (after propensity score matching).

Variable	Proportion among Individuals with CRC (%) *N* = 15,619	Proportion among Individuals without Cancer (%) *N* = 78,095	*p* Value *
Age (Mean, SD)	68.8 (12.4)	68.8 (12.4)	0.889
Age ≤50	1219 (7.8)	6085 (7.8)	0.999
Age 51–60	2471 (15.8)	12,347 (15.8)	
Age 61–70	4036 (25.8)	20,176 (25.8)	
Age 71–80	5325 (34.1)	26,586 (34.0)	
Age >80	2568 (16.5)	12,901 (16.5)	
Women	6933 (44.4)	34,774 (44.5)	0.749
Men	8686 (55.6)	43,321 (55.5)	
2005–2010	3782 (21.0)	16,320 (20.9)	
2011–2016	5882 (37.7)	29,367 (37.6)	0.912
2017–2022	6455 (41.3)	32,408 (41.5)	
Number of physician visits per year during the follow-up (Mean, SD)	8.2 (4.5)	8.2 (4.5)	0.809
Charlson comorbidity score (Mean, SD)	2.4 (2.0)	2.4 (2.0)	0.789

Proportions of patients in given as %, unless otherwise indicated. SD: standard deviation. * Wilcoxon signed-rank, McNemar, and Stuart–Maxwell tests; all standardized mean differences: <0.1.

**Table 2:** Association between CRC and subsequent psychiatric diagnosis in patients followed in general practices in Germany by period (univariable Cox regression models).

Patients Group	2005–2010	2011–2016	2017–2022
	HR (95% CI)	HR (95% CI)	HR (95% CI)
Any psychiatric disorder	1.64 (1.41–1.92) *	1.86 (1.68–2.08) *	1.90 (1.72–2.10) *
Depression	1.73 (1.45–2.07) *	1.93 (1.70–2.19) *	2.08 (1.83–2.36) *
Anxiety	1.86 (1.24–2.79) *	1.81 (1.40.2.35) *	2.59 (2.11–3.17) *
Reaction to severe stress and adjustment disorder	1.31 (0.91–1.89)	1.67 (1.36–2.05) *	1.55 (1.31–1.84) *

* *p* < 0.01.

## Data Availability

The data that support the findings of this study are available from the corresponding author on reasonable request.
